# The Effects of Birth Year, Age and Sex on Hemagglutination Inhibition Antibody Responses to Influenza Vaccination

**DOI:** 10.3390/vaccines6030039

**Published:** 2018-07-03

**Authors:** Ewan P. Plant, Angelia A. Eick-Cost, Hussein Ezzeldin, Jose L. Sanchez, Zhiping Ye, Michael J. Cooper

**Affiliations:** 1 Division of Viral Products, Center for Biologics Evaluation and Research, US Food and Drug Administration, Silver Spring, MD 20903, USA; zhiping.ye@fda.hhs.gov; 2 Armed Forces Health Surveillance Branch, Public Health Division, J3/CSA, Defense Health Agency, and Cherokee Nation Technology Solutions, Silver Spring, MD 20904, USA; angelia.a.cost.ctr@mail.mil (A.A.E.-C.); jose.l.sanchez76.civ@mail.mil (J.L.S.); michael.cooper3@nih.gov (M.J.C.); 3 Office of Biostatistics and Epidemiology, Center for Biologics Evaluation and Research, US Food and Drug Administration, Silver Spring, MD 20903, USA; hussein.ezzeldin@fda.hhs.gov

**Keywords:** influenza, serum, IgG, humoral antibody, original antigenic sin, hemagglutinin

## Abstract

The first exposure to influenza is thought to impact subsequent immune responses later in life. The consequences of this can be seen during influenza epidemics and pandemics with differences in morbidity and mortality for different birth cohorts. There is a need for better understanding of how vaccine responses are affected by early exposures to influenza viruses. In this analysis of hemagglutination inhibition (HI) antibody responses in two cohorts of military personnel we noticed differences related to age, sex, prior vaccination, deployment and birth year. These data suggest that HI antibody production, in response to influenza vaccination, is affected by these factors. The magnitude of this antibody response is associated with, among other factors, the influenza strain that circulated following birth.

## 1. Introduction

Inactivated influenza vaccines have been widely shown to reduce the morbidity and mortality associated with influenza. The level of hemagglutination inhibition (HI) antibodies correlated with protection has been established in several studies [1,2,3,4]. However, HA (Hemagglutination Assay) antibody levels to specific viruses don’t afford broad protection and have been shown to wane after vaccination [5]. Regular seasonal vaccination is known to boost HI antibodies to the vaccine strains but the observed increase is reduced with additional vaccinations and age [6,7,8,9]. In general, the time taken for these antibodies to decrease to levels not associated with protection has not been well studied. Some studies have indicated the benefits of vaccination can extend into the following season [5,10,11,12,13,14] while another study indicated that such antibody levels drop below accepted protective levels within a year [15]. Measurements of HI titers 180 days post-vaccination suggest that a significant portion of vaccinated subjects retain protective titers up to this point [16], but it is known that a reduction in vaccine effectiveness occurs later in the season, or in between seasons [17,18,19,20]. This has implications for those travelling between the northern and southern hemispheres, military personal posted to different regions of the globe, and at risk people vaccinated early during a season when influenza virus infections emerge late in the season.

Some studies, with sex-stratified data have found that women develop higher HI antibody levels toward some influenza vaccine antigens following vaccination [21]. Higher titers have been reported for women in subjects that seroconverted after the emergence of the 2009 pandemic H1N1 strain [15]. However, after six months the antibody levels were similar between the male and female populations. In a previous analysis using Department of Defense Serum Repository (DoDSR) samples we observed that the antibody titers between two weeks and six months after vaccination were often lower for women [22]. It was not clear if this was due to unmeasured confounders related to the cohort or to actual sex differences in antibody waning.

It has been reported that antibody titers toward the first influenza strains encountered early in life are boosted by subsequent infections with antigenically drifted viruses (reviewed in [23]). In contrast to the anamnestic response to a previously encountered strain, serum from the secondary response to an antigenically drifted strain is more cross-reactive [24]. There is evidence that infection, or vaccination with a new strain, provides a significant boost to pre-existing antibodies levels [22,25,26]. It is thought that the antigenic distance between the strains also affects the generation of the antibody response [27]. The impact that circulating influenza viruses experienced during early childhood have on subsequent risk of infection and antibody responses are documented [23,28,29], but the impact of prior immunity to circulating influenza viruses on vaccine response is not known; and this is a critical question that needs to be investigated for the development of universal vaccines [30].

The initial focus of this study was to investigate antibody waning in men and women after influenza vaccination. Antibody titers, two to six weeks post-vaccination, towards the A(H1N1), A(H3N2), and B influenza virus strains included in the vaccine, were assessed by HI assay. Titers from a second blood draw taken during the following year were also measured. Analyses of factors that might affect the antibody response, such as prior vaccination or infection, year of birth, preparation for deployment, and length of time in military service, were assessed for impact upon HI titer. The results led us to hypothesize that influenza viruses encountered in the first years of life affect vaccine responses later in life in a strain specific manner.

## 2. Materials and Methods

The study population consisted of active component service members who received an inactivated trivalent influenza vaccine in 2003, 2010, or 2011. The vaccination was required to occur between 1 September and 15 December of the year of interest, and the individuals could only receive one influenza vaccination per year. To reduce potential confounders, service members were excluded if their length of time in the military was greater than 10 years before the post-vaccination sample, or if they had a medical encounter for an influenza-like illness (ILI) or pneumonia and influenza (PI) for the time period of the study.

Serum samples were obtained from the Department of Defense Serum Repository (DoDSR), maintained by the Armed Forces Health Surveillance Branch of the Defense Health Agency, in accordance with the relevant guidelines and regulations. The first serum collection window was two to six weeks after receipt of the influenza vaccination. The second serum sample was collected during the year following the first sample. Three different bins were used to define the window for the second serum collection; 20% of samples were 3–4 months post-vaccination, 30% were 5–7 months post-vaccination, 40% were 8–10 months post-vaccination, and 10% were 11–12 months post-vaccination. The rationale for selecting more samples 5–10 months post-vaccination was based on publications indicating that protective HI titers were maintained up to 6 months before dropping to non-protective levels [15,16].

The WHO recommendations for influenza vaccine formulation for the Northern hemisphere from 1990 forward were analyzed. Consecutive seasons with no strain changes were selected for additional inspection. This allowed us to expand the possible pool of available subjects and to reduce confounding effects associated with a specific season. The Northern hemisphere influenza season spans two calendar years but, as the subjects were all vaccinated at the end of the first calendar year, the groups are referred to by that year. Two groups, each comprised of 80 female and 80 male subjects, were selected. The first group was vaccinated in 2003; the second group was vaccinated in 2010 or 2011 (see Table 1). The female and male groups were matched, where possible, with regard to the timing of the blood draws.

Hemagglutination inhibition (HI) assays were performed using standard methods. Each volume of serum was treated with 3 volumes of receptor destroying enzyme (Accurate Chemical, Westbury, NJ, USA) at 37 °C overnight, which was then inactivated by heating in a 56 °C water bath for 30 min. Six volumes of PBS were added to make the final sera concentration of 1:10. After RDE treatment, 50 microliters of each diluted serum was added to the first well of a 96-well plate. 25 microliters of PBS was added to the next 11 wells and serial 1:2 dilutions of the sera were made using robotic diluters. 25 microliters of virus titrated to a final HA titer of 4 was added to the first 11 wells and the plates incubated at room temperature for 30 min. The last well was left as a no-sera control. Freshly diluted turkey red blood cells (0.5%) for H1N1 and B antigens, or guinea pig red blood cells (1.0%) for H3N2 antigens, were added to all wells and the plates incubated for an additional hour. After incubation, the last dilution of sera that completely inhibited agglutination was recorded. Raw data is available in the Supplementary Materials.

Anti-human IgG Quantitation (AHQ) dip and read biosensors were used with a Pall ForteBio Octet QKe system to measure the relative quantity of IgG in the sera samples. A standard curve was made using dilutions of a sera sample that gave a high read and the samples tested were measured relative to that. Samples were measured in duplicate and averaged.

Geometric mean titers (GMT) were calculated from the log values of the HI titers. Univariate analyses were performed using GraphPad Prism 6. Unless noted otherwise, the data for age, time of sample collection, HI titers and time in service were rank ordered and the Pearson correlation coefficients were calculated in Excel for correlation analyses. The R package, nlme [31] was used to conduct stepwise multivariate regression analyses to explore the independent effects of sex, age, years in service, deployment status, prior vaccination, and race on the post-vaccination response. A linear-mixed model (LMM) was used to study these independent effects on the post-vaccination response to the H1N1, H3N2, and B antigens for the 2003 and 2011 cohorts. The LMM (fit using restricted maximum likelihood) included random effect for sex, years of service, deployment status and hierarchical effects for age within years of service (scripts available upon request).

## 3. Results

### 3.1. Study Population

The study subjects are from a highly vaccinated active component service member population with near complete capture of medical encounters. Serum samples from military personnel are routinely collected upon entry to military service and at other times such as before and after deployment. Subjects identified for the study had a blood draw 2–6 weeks after seasonal influenza vaccination (mean of 31.7 days with a standard deviation of ±6.2 days), and a second blood draw later that year. Because the ratio of men to women is higher in the military, samples from women were identified first, followed by samples from men matched for timing of the second blood draw and age. The age of the subjects ranged from 18 to 41 at the time of vaccination with an average of 26.7 years (standard deviation of ±5.5). The race was predominantly White (53%), followed by Black (25%), Hispanic (12%), Asian/Pacific Islander (3%), Other (3%), American Indian/Alaskan Native (2%), and Unknown (2%). Two cohorts of subjects vaccinated during different influenza seasons were identified to maximize the available sera and to reduce possible confounders associated with a particular season. The first cohort was vaccinated in 2003 and all but six members of the second cohort were vaccinated in 2011. These members included three men and three women; each vaccinated in 2010. The influenza vaccine strains did not change from 2010 to 2011. The years each cohort was vaccinated, and the strains included in the vaccines, are presented in Table 1.

### 3.2. Comparison of HI Titers for Males and Females

Geometric mean titers (GMTs) were compared between men and women for samples drawn 2–6 weeks post-vaccination. There was a trend of lower GMTs for vaccine antigens among women, with the exception of the H3N2 antigen in the 2011 cohort (Table 2 and Table 3). The difference in post-vaccination titers did not reach statistical significance (Mann Whitney rank test).

**Figure 1:**
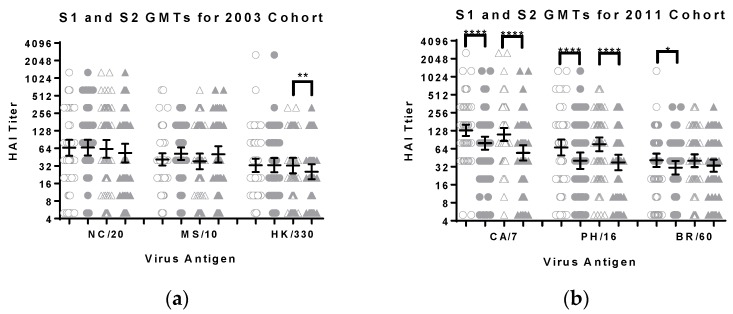
The geometric mean titers, with 95% confidence intervals, for sera samples from the (**a**) 2003 and (**b**) 2011 cohorts are shown. The individual S1 and S2 data points are shown as open and filled symbols respectively, circles for men and triangles for women. The antigens tested with the 2003 cohort were A/New Caledonia/20/1999 (H1N1), A/Moscow/10/1999 (H3N2) and B/Hong Kong/330/2001 (Victoria lineage). The antigens tested with the 2011 cohort were A/California/07/2009 (pdmH1N1), A/Perth/16/2009 (H3N2) and B/Brisbane/60/2008 (Victoria lineage). The difference between male and female titers (S1 or S2) did not reach the level of significance (Mann Whitney test). The difference between the S1 and S2 titers for each group was evaluated using the Wilcoxon matched-pairs rank test and the *p*-values are shown: *, *p* ≤ 0.05; **, *p* ≤ 0.01; and ****, *p* ≤ 0.0001.

Changes in titers between the first and second serum samples were also compared. There was a significant (*p* < 0.0001, Wilcoxon matched-pairs rank test) decrease in GMTs for the influenza A viruses among men and women in the 2011 cohort (Figure 1). The decrease was also significant for the influenza B virus for men (*p* < 0.05, Wilcoxon matched-pairs rank test) but not for women in the 2011 cohort. There were no significant decreases in titers for men in the 2003 cohort but there was a significant decrease in titers toward the B virus for women in the 2003 cohort (*p* < 0.01, Wilcoxon matched-pairs rank test) (Figure 1). There were increases in GMT for the H3N2 antigen for both men and women in the 2003 cohort, but these did not reach a level of statistical significance. Further inspection of the raw data revealed that many of the subjects had higher titers in the second blood draw, suggesting an intervening infection or variability in the assay. The titer from the second blood draw was lower for more female than male subjects (Table 2 and Table 3). Therefore, the data were reanalyzed including only subjects with lower titers in the second blood draw. The trend for lower GMTs for women toward the vaccine antigens remained with the exception of the influenza B strains (data not shown).

The antibody decline over time for each of the cohorts was also evaluated (see Supplementary Materials Figure S1). The GMT values for the 3–4, 5–7, 8–10 and 11–12 months data points were determined for each cohort. The initial decline in HI titer for both of the influenza A viruses in the 2011 cohort subjects was greater than that for the influenza B antigen. This pattern was not observed for the 2003 cohort in which there was an increase in GMT values for the 3–4 months data points and less decline overall. This suggests differences in rate of decline depend on the antigen and the season. The pattern of decline was similar for men and women in the respective seasons with one exception. In the 2003 cohort, the titers 11–12 months after vaccination increased for men for all three antigens, but not for women. However, the number of subjects in these groups was small and the difference between men and women was not found to be statistically different (Mann Whitney rank test).

### 3.3. Age

It is known that HI titers post-vaccination negatively correlate with increasing age [6,7], even within a young adult population [22]. There were negative correlations between age and HI titer for most antigens (Table 4). 

The negative correlations were statistically significant (Spearman rank correlation coefficient in Excel) for all antigens for men in the 2003 cohort but only for the H1N1 antigen for women. The negative correlations reached significance for only one antigen in the 2011 cohort, the A(H3N2) antigen for women. There were positive correlations between age and HI titer for the B antigen for both sexes in the 2011 cohort but it only reached significance for men. Analysis of each cohort using a linear-mixed model (LMM) revealed a negative correlation between age and HI titer for the influenza A antigens in the 2003 cohort with sex having minimal effect. In the 2011 cohort there was a positive correlation between age and HI titer for the B antigen with sex having a minimal effect. 

Negative correlations between HI titer and age were also observed with the samples collected later in the year. In the 2003 cohort, significant negative correlations were observed for all three antigens for men but only for the A(H1N1) antigen for women. The negative correlation for the A(H1N1) antigen reached statistical significance for men in the 2011 cohort. The correlation between age and titer for the B antigen was positive for both men and women in the 2011 cohort.

### 3.4. Prior Seasonal Vaccination

Medical records indicated that many of the subjects had received an influenza vaccination the year prior to the study. Approximately 40% of men and 35% of women in the 2003 cohort had received seasonal influenza vaccinations in 2002. In the 2011 cohort, 81% of men and 64% of women received seasonal vaccinations in the prior season. Subjects in the 2011 cohort could have received different types of influenza vaccines. The CVX codes for prior season vaccination differentiated between Live Attenuated Influenza Vaccines (LAIV) and Inactivated Influenza Vaccines (IIV) allowing for a more refined analysis. Analyses on the S1 data, blood drawn 2–6 weeks after vaccination, were performed to determine if there were any detectable effects of vaccination the prior season, by type, on HI titers.

**Figure 2:**
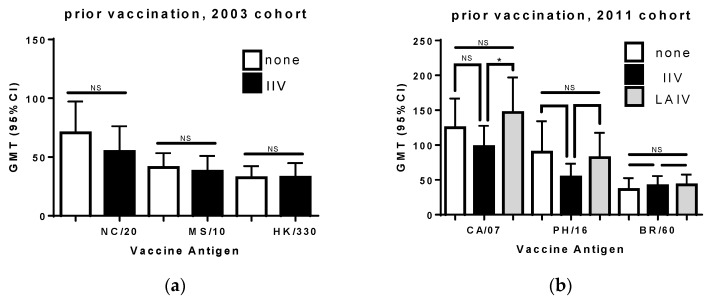
The geometric mean titers (with 95% confidence interval) for previously unvaccinated subjects are compared to titers of those with records of vaccination the previous season. Results are shown for the (**a**) 2003 and (**b**) 2011 cohorts. The viruses used for testing HI titer are listed on the *x* axis: NC/20, A/New Caledonia/20/1999 (H1N1); MS/10, A/Moscow/10/1999 (H3N2); HK/330, B/Hong Kong/330/2001 (Victoria lineage); CA/7, A/California/07/2009 (pdmH1N1); PH/16, A/Perth/16/2009 (H3N2); and BR/60, B/Brisbane/60/2008 (Victoria lineage). The difference between the previously vaccinated group and those vaccinated in the current season was evaluated using the Mann Whitney rank test. n.s., not significant; and *, *p* < 0.05.

Analysis of the 2003 cohort showed that the HI titers for the A(H1N1) antigen were slightly lower in subjects that had been vaccinated the prior year (Figure 2). This was observed for both men and women but was not found to be statistically significant (Mann Whitney test rank test). 

Analysis of the 2011 cohort indicated that prior vaccination with IIV resulted in lower HI titers toward both the A(H1N1) and A(H3N2) components of the vaccine. This difference was not statistically significant (Mann Whitney rank test). In contrast, the titers for those vaccinated with LAIV the prior year were similar to, or higher than, the titers of those who were not vaccinated the prior year. The GMTs for the A(H1N1) strain was significantly higher than the titers for those vaccinated with an IIV (*p* < 0.05, Mann Whitney rank test). There was no difference in the influenza B titers regardless of prior seasonal vaccination status, reflecting the higher conservation of influenza B HA, and perhaps a higher incidence of cross-reactive antibodies [32]. These results are in line with other studies indicating that individuals who have been vaccinated previously have lower titers than those only vaccinated in the current season, unless the prior vaccination was an LAIV [6,7,33,34,35].

**Figure 3:**
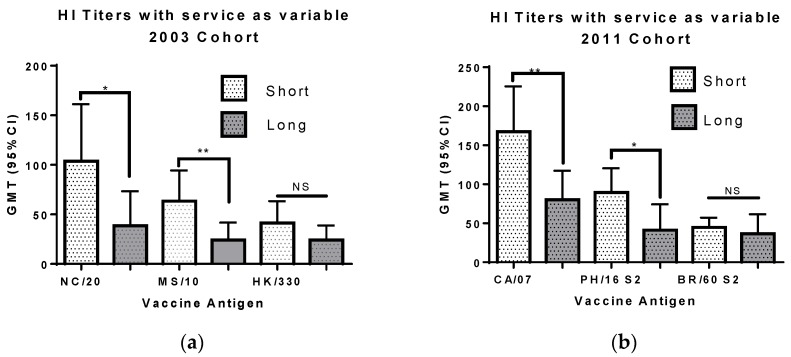
The geometric mean titers (with 95% confidence interval) for sera from personnel with short and long service are shown. The data for (**a**), the 2003 cohort, are from 48 subjects with 1–2 years of service and 19 subjects with 8–9 years of service. The data for (**b**), the 2011 cohort, are from 63 subjects with 1–2 years of service and 23 subjects with 8–9 years of service. The viruses used for testing HI titer are listed on the x axis: NC/20, A/New Caledonia/20/1999 (H1N1); MS/10, A/Moscow/10/1999 (H3N2); HK/330, B/Hong Kong/330/2001 (Victoria lineage); CA/7, A/California/07/2009 (pdmH1N1); PH/16, A/Perth/16/2009 (H3N2); and BR/60, B/Brisbane/60/2008 (Victoria lineage). The difference between the short and long service groups was evaluated using the Mann Whitney rank test. NS, not significant; *, *p* < 0.05; and **, *p* < 0.01.

Receipt of multiple annual influenza vaccinations is known to blunt the HI antibody response [6,7,8]. Our study protocol only included vaccination history for the prior year (see Methods section). Because the military has strict vaccination requirements we used the length of military service as a proxy for multiple vaccinations. Sera from men and women in the 2003 cohort who had been in military service for 8–9 years were compared to those who had been in military service for only 1–2 years (Figure 3). In this cohort, subjects with a shorter time in the service had higher titers toward all three antigens, the difference being significant for the A(H1N1) and A(H3N2) antigens (*p* < 0.05 and *p* < 0.01 respectively, Wilcoxon matched-pairs rank test). 

Sera from men and women in the 2011 cohort who had been in military service for 8–9 years were compared to those who had been in military service for only 1–2 years (Figure 3). In this cohort, subjects with a shorter time in the service had higher titers toward all three antigens, the difference being significant for the A(H1N1) and A(H3N2) antigens (*p* < 0.01 and *p* < 0.05 respectively, Wilcoxon matched-pairs rank test). Analysis of each full cohort using LMM revealed a negative correlation between age and years in service for both cohorts. In the model the HI titers toward both influenza A antigens were explained by age for the 2003 cohort and years in service for the 2011 cohort. The impact of each variable was significant for the influenza B antigen in the 2011 cohort but neither variable had a significant effect on the 2003 cohort HI titers.

A subset of samples from the 2011 cohort was analyzed for HI titers toward the older antigens. Sera from 12 men and 12 women from the 2011 cohort that had been in military service 8–9 years were compared to sera from personnel, of a similar age, who had been in military service for only 1–3 years (see Supplementary Materials Figure S2). Lower titers were observed for the subset with longer military service, but the differences did not reach statistical significance. In contrast, when the HI titers toward older antigens were measured, the titers were higher for personnel that had served longer (see Supplementary Materials Figure S2). This result, referred to as antigenic seniority, is similar to other reports describing an increase in antibody titer toward previously encountered antigens after infection or vaccination [25,26,36,37]. Total IgG levels in the samples in the 2011 service subset were compared. The GMTs were similar for the shorter and longer military service groups. However, when the samples were stratified by sex, the IgG titers for women were found to be significantly lower than those of men (see Supplementary Materials Figure S3). There was no correlation between IgG levels and age, or timing of the blood draw (Spearman test in prism).

### 3.5. Deployment

**Figure 4:**
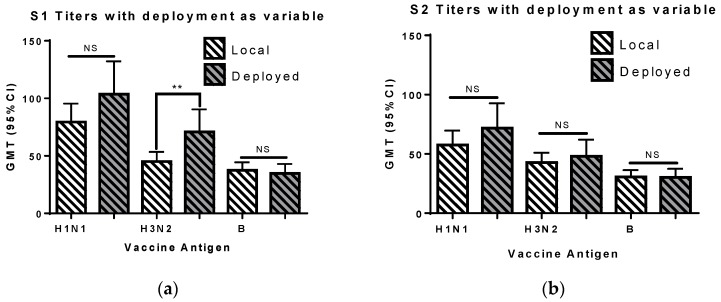
The geometric mean titers (with confidence interval) with deployment as a variable is shown for the S1 and S2 blood draws. The viruses used for testing HI titer for 2003 cohort subjects were A/New Caledonia/20/1999 (H1N1), A/Moscow/10/1999 (H3N2), and B/Hong Kong/330/2001 (Victoria lineage). The viruses used for testing HI titer for 2003 cohort subjects were A/California/07/2009 (pdmH1N1), A/Perth/16/2009 (H3N2), and B/Brisbane/60/2008 (Victoria lineage). The difference between the two groups was evaluated using the Mann Whitney rank test. NS, not significant; and **, *p* < 0.01.

We analyzed deployment as a variable. Approximately 38% of the 320 subjects (60 men and 60 women) were deployed between the time of vaccination and the final blood draw (Figure 4). The mean age for the deployed (26.4 years, standard deviation of ±5.4) and non-deployed (26.8 years, standard deviation of ±5.5) groups was similar. The GMT value for the first blood draw was significantly higher for the deployed personnel compared to the non-deployed group (Mann Whitney rank test). The A(H3N2) titers remained higher in deployed subjects when different sub-populations were analyzed. The difference was significant for the 2011 A(H3N2) antigen (A/Perth/16/2009), but not for the 2003A(H3N2) antigen (A/Moscow/10/1999), when the 2003 and 2011 cohorts were analyzed separately. The difference remained significant for men, when the two sexes were analyzed separately, but not for women. There was no significant difference in the HI titers in samples collected later in the year between subjects who were or were not deployed (Figure 4b). These results suggest that something associated with the preparation for deployment primes the immune system, and results in greater production of antibodies toward the A(H3N2) vaccine component in the 2–6 weeks following vaccination.

### 3.6. Year of Birth

In general, post-vaccination GMTs decreased gradually with increasing military service (Figure S4). However, there were two notable exceptions. First, in the 2003 cohort, the GMTs for the A(H1N1) antigen were noticeably higher for those with 1–4 years of military service compared to those with 5–8 years. This was not apparent for the A(H3N2) and B antigens. The birth years for the subjects in the 2003 cohort flanked the emergence of an A(H1N1) strain in 1977 after a 20-year absence. Second, the GMT for the A(H1N1) antigen in the 2011 cohort was highest in those with two years of service. This suggests something prior to military service reduced the response to vaccination for those in the first year of service, or something during the initial year of service, such as vaccination or exposure to the 2009 pdmH1N1 strain, increased responses for those already in military service. Interestingly, the titers for the A(H1N1) antigen increased from three to five years of service while the titers for the B antigen decreased over this time. This indicated that the trends were unique for each antigen.

It is hypothesized that the first exposure to an influenza virus influences the later response of the immune system. This effect is often referred to as antigenic sin [23,28,29]. The A(H1N1) viruses began circulating in 1977 after a 20-year absence. Approximately one half of the subjects in the 2003 cohort were born before 1977. The GMT toward the A(H1N1) vaccine component of those born before 1977 were compared to those born after 1977. The titers for those born after 1977 were significantly higher (Mann Whitney rank test, *p* < 0.0001) suggesting that exposure to an A(H1N1) virus early in life resulted in a more robust antibody production with A(H1N1) vaccination later in life (see Supplementary Materials Figure S5).

The effect of antigenicity of viruses circulating early in life on vaccine response was evaluated. Data was analyzed by the year the subjects were born and the viruses known to be circulating during the first years of life. The subjects were grouped by age, binning for each year of age from 19 to 29 separately for both the 2003 and 2011 cohorts. Each of these bins contained results for 6–13 subjects. The geometric mean titers were determined for each age group and graphed (see Supplementary Materials Figure S6). We used information from the WHO vaccine strain recommendations, MMWR (Morbidity and Mortality Weekly Report) reports, and other publications [38,39,40,41] to identify the dominant circulating strains and severity of the influenza season for each year considered (Table 5 and Table 6). The years in which the vaccine strains remained the same were used as an indicator that the circulating strains remained antigenically similar (vertical lines in Figure S6).

**Figure 5:**
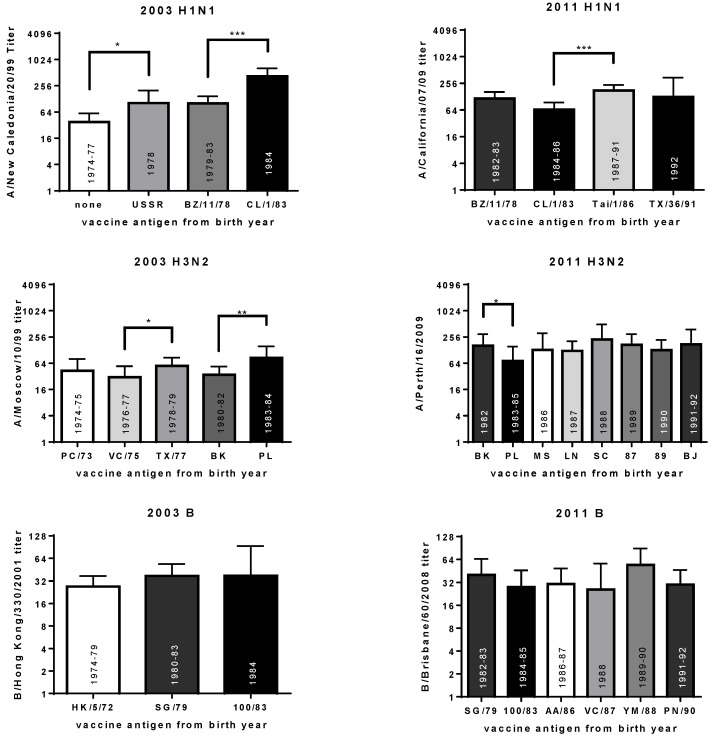
The geometric mean titers (with confidence interval) towards the vaccine antigens for the 2003 and 2011 cohorts based on antigens circulating after the year of birth. The year the antigens were recommended as vaccine strains are indicated inside the bars. The difference between sequential groups, born when different viruses circulated, was evaluated using the Mann Whitney rank test. *, *p* < 0.05; **, *p* < 0.01; and ***, *p* < 0.001. The viruses used for HI titer are A/New Caledonia/20/1999 (H1N1), A/Moscow/10/1999 (H3N2), and B/Hong Kong/330/2001 (Victoria lineage) for the 2003 cohort. The viruses used for testing HI titer are A/California/07/2009 (pdmH1N1), A/Perth/16/2009 (H3N2), and B/Brisbane/60/2008 (Victoria lineage) for the 2011 cohort. The H1N1 strains from the birth year are USSR (A/USSR/90/1977), BZ/11/78 (A/Brazil/11/1978), CL/1/83 (A/Chile/1/1983), Tai/1/86 (A/Taiwan/1/1986) and TX/36/91 (A/Texas/36/1986). The H3N2 strains from the birth year are PC/1/73 (A/Port Chalmers/1/1973), VC/3/75 (A/Victoria/2/1975), TX/1/77 (A/Texas/1/1977), BK (A/Bangkok/1/1979), PL (A/Philippines/2/1982), MS (A/Mississippi/1/1985), LN (A/Leningrad/360/1986), SC (A/Sichuan/2/1987), 87 (A/Shanghai/11/1987), 89 (A/Shanghai/16/1989), and BJ (A/Beijing/353/1989). The B antigens from the birth year are HK/5/72 (B/Hong Kong/5/1972), SG/79 (B/Singapore/222/1979), 100/83 (B/USSR/100/1983), AA/86 (B/Ann Arbor/1/1986), VC/87 (B/Victoria/2/1987), YM/88 (B/Yamagata/16/1988) and PN/90 (B/Panama/45/1990).

The results from individuals born in seasons with antigenically similar viruses were grouped and GMTs calculated (Figure 5). Statistical analyses were performed to determine if the titers for each group were significantly different from the prior group (Mann Whitney rank tests). As expected, the GMT towards the A/New Caledonia/20/1999 (H1N1) antigen was lower for those born in 1977 or earlier, than those born in 1978 in the 2003 cohort (*p* < 0.05, Figure 5). There was no significant difference in titer for those born when the A/USSR/90/1977 and A/Brazil/11/1978 viruses circulated. However, the GMT for those born when the A/Chile/1/1983 strain circulated was significantly higher than the GMT for those born when the A/Brazil/11/1978 virus circulated (*p* < 0.001). These data suggest that the antigenicity of influenza viruses circulating early in life affect vaccine response.

There were other instances where the titer toward the influenza A vaccine antigens was significantly different after birth year was taken into consideration (that is, the antigenicity of the viruses circulating the year after birth changed) (Figure 5). The change in titer depended on the cohort being analyzed. Subjects from both the 2003 and 2011 cohorts were born during the period when the A/Brazil/11/1978 or A/Chile1/1983 viruses circulated (but the subjects from the 2011 cohort were 8 years older when they were vaccinated than those in the 2003 cohort). While the titers toward the A/New Caledonia/20/1999 (H1N1) vaccine antigen were significantly higher in the subjects born when the A/Chile/1/1983 strain circulated in the 2003 cohort, the titers toward the A/California/07/2009 (pdmH1N1) vaccine antigen were slightly lower, but not significantly different, in the 2011 cohort (Figure 5). A similar pattern was observed for the A(H3N2) antigens. In the 2003 cohort, the GMT toward the A/Moscow/10/1999 vaccine antigen was significantly higher for those born when the A/Philippines/2/1982 virus circulated compared to those born when the A/Bangkok/1/1979 virus circulated. Conversely, the GMT toward the A/Perth/16/2009 vaccine antigen was significantly higher for those born when the A/Bangkok/1/1979 virus circulated in the 2011 cohort. These results suggest a role for age and/or the vaccine antigen, in addition to the antigenicity of the virus circulating after birth.

HI data in the context of which type of virus circulated after the year of birth, and whether the season was mild or severe, was also evaluated. There were several seasons where a particular type of virus circulated (© symbol in Figure S6) and it was described as a severe influenza season (darker shading of bars in Figure S6). Many of these seasons correlated with higher titers after vaccination (for example: A(H1N1) viruses in 1978 and 1983, A(H3N2) viruses in 1980 and 1984, and B viruses in 1976 and 1979 for the 2003 cohort; A(H3N2) viruses in 1982, 1987, 1989 and 1991, and B viruses in 1985 and 1990 for the 2011 cohort). However, as mentioned above, this effect also depended on the vaccine antigen being tested: A(H3N2) titers were low in the 2003 cohort for subjects born in 1982 and high for those born in 1984, but this pattern was reversed in the 2011 cohort (Figure 5). This effect could also be due to the age of the subject at the time of vaccination (subjects born in 1984 were 19 years old in the 2003 cohort, or 27 years old in the 2011 cohort). The statistical power associated with these statistics is also affected by sample size. For example, when we compared the GMTs toward the H1N1 antigen for those born before and after 1977, a statistical test yielded a *p*-value of <0.0001 (see Supplementary Materials Figure S5). When the subjects born before 1974 and after 1978 were removed from the analysis the *p*-value rose to <0.05 (Figure 5).

## 4. Discussion

This study contributes to existing knowledge about how age and sex affect the response to influenza vaccination. In addition, the analyses reveal correlations between the HI titer after vaccination and other variables. An increase in antibody response among personnel that were deployed after vaccination was observed. This suggests that something in the preparation for deployment better primed those personnel for vaccination. Subclinical infections prior to deployment are one possible explanation but, while MMWR reports indicate the H3N2 viruses circulated at that time [44,45], there could be other factors, such as concomitantly administered vaccines, we have not considered. Analysis of the data presented here, in the context of other variables such as prior vaccination and year of birth, have led us to hypothesize that influenza infections early in life have an impact on vaccine response later in life.

Interest in the impact of sex on clinical trials has increased in recent years [46]. The effect sex has in the response towards influenza infection has been reviewed [47,48], but fewer publications investigate the impact sex has on antibody response to influenza vaccination [21,49,50,51]. There is also a dearth of information about the impact sex may have on the rate of HI antibody waning between men and women. In some instances, a stronger decline in antibody concentrations has been observed in women [52]. Here, as in our previous study [22], higher GMTs for men toward most of the vaccine antigens are reported. This trend did not reach the level of statistical significance and contrasts with some published research for influenza vaccinations [49,53]. It may be due to some aspect of military service, as has been reported for other vaccines [54]. Alternatively, as demonstrated in the literature, the trends could be antigen or season specific. For example, Furman et al., did not observe a difference between the sexes by HI titer for the A(H1N1) antigen, but Hsu et al., observed a difference for the pdmH1N1 antigen [15,55]. The blood draws in our previous study occurred up to six months after vaccination, prompting an assessment of the differences in antibody waning in this study. However, the data from this study, with blood draws between two and six weeks after vaccination, still indicated a trend for lower titers in young adult women. Additionally, there was a difference in the amount of total IgG between the sexes. A greater number of blood samples collected from women later in the year showed a decrease in HI titer than those collected from men. These results leave questions about sex based differences in antibody levels, IgG concentrations and HI antibody waning after influenza vaccination unanswered. More research in this area is required to shed light on these issues.

A major limitation of this study design is the reliance on historic blood samples rather than designing a clinical trial with fixed sample collection times. This meant that antibody levels were not assessed at multiple time points from individuals throughout the year. Only two samples were collected from each subject and the timing of sample collection varied. Subjects were binned based on the second blood draw. This clustering may have hindered the detection of differences in the rate of antibody waning between men and women. This disadvantage was offset by the ability to include larger cohorts and compare different seasons. This study describes other effects, such as the effect of prior vaccination, without season-specific confounders. Complete vaccination records were not available for these analyses so “length of service” was used as a proxy for vaccination exposure in our analyses. Given the DoD vaccination policy regarding influenza vaccination and the relatively sparse uptake of influenza vaccine for young, healthy civilians during the study period, this seems reasonable. This approach, however, could bias our findings if the subjects in these analyses systematically, by study group, were vaccinated for influenza prior to their military service. We feel this is unlikely but cannot conclusively rule it out.

Another limitation of the study was the low number of subjects born in particular years which limited the statistical rigor of our analysis of year-of-birth effects. For the analysis of birth cohorts born before and after the emergence of the A(H1N1) viruses in 1977, the *p*-value dropped from <0.0001 to <0.05 when the titer of single year birth cohorts were compared (see Supplementary Materials Figure S5 and Figure 5). However, even with the limited number of subjects in each birth cohort, significant differences were observed supporting the hypothesis that birth year has an impact on vaccine response. Another limitation of this work is that, for ease of analysis, we assumed that the subjects were likely exposed to influenza in the first year of life. The majority of childhood influenza infections likely occur in the first 5 years rather than the first year. In addition, we assume the subjects of the same birth cohort were likely exposed to the same viruses in childhood but they may have been exposed to quite different viruses depending on their geographical location. Our approach relies on an analysis of a population, and as such, is subject to the limitations of sampling.

The effect that birth year has on susceptibility to influenza disease, and for vaccine effectiveness, is of great interest [20,23,56,57]. In addition to factors related to an aging immune system, it appears that exposure to particular viral serotypes in the years immediately after birth also affects a person’s response to vaccination [58]. In a recent study by Skowronski et al., they noted lower vaccine efficacy for the pdmH1N1 virus in the birth cohort for the years 1957 to 1967, that were likely primed with an A(H3N2) antigen rather than an A(H1N1) antigen [20]. Similarly, lower A(H1N1) antibody titers in subjects born prior to the re-emergence of A(H1N1) viruses in the late 1970s were observed in this study. In another study, Skowronski et al. reported differences in the percentage of B/Victoria and B/Yamagata infections by year of birth [59]. When information about the viruses that circulated following the birth year of the subjects in this study was included, we observed differences in HI antibody titers that appear to be due to the antigenicity of the virus circulating in the first years of life, not just the presence or absence of a virus. These differences support the notion that the environment plays a significant role in priming and shaping the immune response [60] and the recommendation that antigenic sin be considered in the development of immunization strategies [23].

There is variability in the amplitude and direction of antibody response after repeat annual seasonal influenza vaccination, and the responses to the influenza B strains differ from the influenza A strains (reviewed in [61]). It has been reported that as pre-seasonal immunity toward influenza B viruses increases, vaccine effectiveness is less impacted by strain mismatches [62]. This is perhaps expected because of the generation of cross-reactive antibodies after influenza B infection [63]. In studies with repeatedly vaccinated individuals it is not clear if the lower subsequent HI titers correlate with vaccine efficacy because other components of the immune response are not measured [32]. This may depend on antigenic distance and the relative abundance of cross-reactive antibodies. In our current analyses, a decrease in HI titers for the pdmH1N1 component in subjects with prior vaccination was observed. Skowronski et al. reported lower vaccine efficacy for pdmH1N1 viruses for subjects that had received both current and prior season vaccination compared to those with just the current season vaccination [20]. This would suggest a correlation between HI titers, prior vaccination, and vaccine efficacy. Skowronski et al., did not observe a difference in efficacy against influenza B for those previously vaccinated and we did not observe a difference in titers for the influenza B component between those with or without prior vaccination. The differences between influenza A and B strains suggest that the HI antibody landscape develops differently for influenza A and B strains and that this difference may affect vaccine efficacy.

Responses to the same influenza vaccine antigen can vary from season to season. The responses to vaccination for a predominantly pediatric population were recorded for 10 years from 1982 until 1992 [64]. In that study, there was variation in vaccine response each year, with a significant increase in influenza B antibodies over time, even when the vaccine antigen did not change. Interestingly, the yearly variation in GMTs for the B component was greater than for the A(H1N1) component, which was greater than for the A(H3N2) component. More peaks and troughs in response to vaccination were observed for the B, H1N1 and H3N2 antigens, respectively, for the 2011 cohort subjects born in those years in this study (see Supplementary Materials Figure S6). Most striking, there are peaks for the B component in 1984–1986 and 1990–1991 in both studies, during seasons that were described as dominantly B seasons. These data suggest that subclinical infections not only boost vaccination responses in children in a contemporary setting, but may also contribute to vaccine responses in a similar manner later in life.

The shaping of the influenza antibody landscape will vary from individual to individual and there are many factors that will contribute to it. High levels of HI antibodies toward the A/California/7/2009 (pdmH1N1) strain in DoDSR serum collected from military service members in 2010, prior to vaccination, were reported indicating high rates of subclinical infection [22]. Also, it has been reported elsewhere that subclinical infections may influence the response to vaccination [65,66]. From the data presented here and elsewhere [35], it is apparent that prior LAIV vaccination can also affect antibody titers after subsequent IIV vaccination. These data support the hypothesis that infections, including subclinical infections, early in life may also play a role in vaccine responses later in life. Recent prior vaccination with an IIV leads to lower vaccine response as measured by HI assay. Year of birth plays a significant role. While others have described differences in vaccine efficacy or mortality [20,56], this study found differences in antibody generation after vaccination. Titers differed not only between birth cohorts, but also within the same birth cohorts receiving vaccinations at different ages. Differences in the intensity of prior influenza seasons that people have been exposed to also appears to have an impact on later vaccine responses. It will be important to determine how these factors will affect responses in universal vaccines that are being developed [30]. Current influenza vaccines are designed to increase antibody titers toward contemporary strains but there is still variability in outcomes depending on many factors including age and prior vaccination status. Better understanding of the effects of antigenic priming on influenza vaccination now will improve the interpretation of HI antibody responses toward universal vaccinations in the future.

## 5. Conclusions 

Our data indicate that several factors influence the antibody response toward influenza vaccination. These include birth year, age at vaccination, sex, prior vaccination experience and preparation for deployment. However, responses are also different toward the different vaccine formulations specific to each season making it difficult to quantify the impact of each of these factors.

## Figures and Tables

**Table 1:** Experimental Cohorts, Virus Vaccine Strains and Years Vaccinated. See Materials and Methods section for more details.

Cohort	Vaccine Strains	Years Vaccinated
2003	A/New Caledonia/20/1999 (H1N1), A/Moscow/10/1999 (H3N2), B/Hong Kong/330/2001 (Victoria)	2003
2011	A/California/7/2009 (pdmH1N1), A/Perth/16/2009 (H3N2), B/Brisbane/60/2008 (Victoria)	2010 or 2011

**Table 2:** HI Data for the 2003 Cohort. The names of the antigens used in the HI (Hemagglutination Inhibition) assay are listed in the first column. The geometric mean titers immediately after vaccination (S1) and later in the year (S2) are shown for each antigen for each group (Men or Women). The *p*-values for significant changes in GMTs between S1 and S2 (Mann Whitney rank test) are indicated by asterisks: **, *p* < 0.01. The number of samples for each group of 80 that had a decrease in titer is shown in the last column.

Antigen	Group	GMT (S1)	GMT (S2)	Number of Samples with Decline
A/New Caledonia/20/1999 (H1N1)	Men	66	66	17
	Women	63	53	30
A/Moscow/10/1999 (H3N2)	Men	41	52	22
	Women	38	51	20
B/Hong Kong/330/2001 (Victoria)	Men	33	33	21
	Women	32	25 **	24

**Table 3:** HI Data for the 2011 Cohort. The names of the antigens used in the HI assay are listed in the first column. The geometric mean titers immediately after vaccination (S1) and later in the year (S2) are shown for each antigen for each group (Men or Women). The *p*-values for significant changes in GMTs between S1 and S2 (Mann Whitney rank test) are indicated by asterisks: ****, *p* < 0.0001; *, *p* < 0.05. The number of samples for each group of 80 that had a decrease in titer is shown in the last column.

Antigen	Group	GMT (S1)	GMT (S2)	Number of Samples with Decline
A/California/07/2009 (pdmH1N1)	Men	130	79 ****	38
	Women	111	55 ****	50
A/Perth/16/2009 (H3N2)	Men	67	40 ****	41
	Women	76	38 ****	53
B/Brisbane/60/2008 (Victoria)	Men	41	31 *	32
	Women	40	33	29

**Table 4:** Correlations between Age and HI Titer. The names of the antigens used in the HI assay are listed in the first column, the 2003 cohort was assayed with the antigens from 1999 to 2001 and the 2011 cohort was assayed with antigens from 2008 to 2009. The Spearman rank correlation coefficient for age and each blood draw, S1 or S2, is shown. The results are in bold where the *p*-values are less than 0.05.

Antigen	Group	S1	*p*-Value	S2	*p*-Value
A/New Caledonia/20/1999 (H1N1)	Men	**−0.541**	**<0.001**	**−0.423**	**<0.001**
	Women	**−0.482**	**<0.001**	**−0.483**	**<0.001**
A/Moscow/10/1999 (H3N2)	Men	**−0.320**	**0.004**	**−0.289**	**0.009**
	Women	−0.183	0.105	−0.127	0.261
B/Hong Kong/330/2001 (Victoria)	Men	**−0.310**	**0.005**	**−0.272**	**0.015**
	Women	−0.009	0.940	0.059	0.604
A/California/07/2009 (pdmH1N1)	Men	−0.213	0.058	**−0.376**	**<0.001**
	Women	−0.105	0.354	−0.160	0.156
A/Perth/16/2009 (H3N2)	Men	0.012	0.915	−0.129	0.252
	Women	**−0.243**	**0.030**	−0.046	0.682
B/Brisbane/60/2008 (Victoria)	Men	**0.267**	**0.006**	**0.257**	**0.016**
	Women	0.027	0.809	**0.236**	**0.035**

**Table 5:** Influenza Vaccine Strain Recommendations from the WHO and Influenza Research Database websites [42,43]. Changes from the previous recommendation are shown in bold.

Year	H1N1	H3N2	B Virus
1974–1975		A/Port Chalmers/1/73	B/Hong Kong/5/72
1975–1976		A/Port Chalmers/1/73	B/Hong Kong/5/72
1976–1977		**A/Victoria/3/75**	B/Hong Kong/5/72
1977–1978		A/Victoria/3/75	B/Hong Kong/5/72
1978–1979	A/USSR/90/77	**A/Texas/1/77**	B/Hong Kong/5/72
1979–1980	**A/Brazil/11/78**	A/Texas/1/77	B/Hong Kong/5/72
1980–1981	A/Brazil/11/78	**A/Bangkok/1/79**	**B/Singapore/222/79**
1981–1982	A/Brazil/11/78	A/Bangkok/1/79	B/Singapore/222/79
1982–1983	A/Brazil/11/78	A/Bangkok/1/79	B/Singapore/222/79
1983–1984	A/Brazil/11/78	**A/Philippines/2/82**	B/Singapore/222/79
1984–1985	**A/Chile/1/83**	A/Philippines/2/82	**B/USSR/100/83**
1985–1986	A/Chile/1/83	A/Philippines/2/82	B/USSR/100/83
1986–1987	A/Chile/1/83	**A/Mississippi/1/85**	**B/Ann Arbor/1/86**
1987–1988	**A/Taiwan/1/86**	**A/Leningrad/360/86**	B/Ann Arbor/1/86
1988–1989	A/Taiwan/1/86	**A/Sichuan/2/87**	**B/Victoria/2/87**
1989–1990	A/Taiwan/1/86	**A/Shanghai/11/87**	**B/Yamagata/16/88**
1990–1991	A/Taiwan/1/86	**A/Shanghai/16/89**	B/Yamagata/16/88
1991–1992	A/Taiwan/1/86	**A/Beijing/353/89**	**B/Panama/45/90**
1992–1993	**A/Texas/36/91**	A/Beijing/353/89	B/Panama/45/90

**Table 6:** The dominant circulating virus type for each northern hemisphere season ending in the years 1973–1993 is shown with references. An additional virus is listed if it contributed to more than 15% of the positive isolates for that season (data from [39].

Year	Dominant Virus Type	References
1973	H3N2	[38,40,41]
1974	H3N2	[38,40,41]
1975	H3N2	[38,40,41]
1976	H3N2	[38,39,41]
1977	B (+25% H3N2)	[38,39,41]
1978	H3N2 (+16% H1N1)	[39,41]
1979	H1N1	[39,41]
1980	B virus	[39,41]
1981	H3N2 (+22% H1N1)	[41]
1982	B virus (+24% H1N1)	[41]; MMWR 1982; 31(27): 375–379
1983	H3N2	[41]; MMWR 1983; 32(29): 373–377
1984	H1N1 (+45% B virus)	[41]; MMWR 1984; 33(29): 417–421
1985	H3N2	[41]; MMWR 1985; 34(28): 440–444
1986	B Virus (+24% H2N3)	[41]; MMWR 1986; 35(29): 475–479
1987	H1N1	[41]; MMWR 1988; 37(31): 466, 468–470, 475
1988	H3N2	[41]; MMWR 1988; 37(32): 497–503
1989	B Virus (+44% H1N1)	[41]; MMWR 1989; 38(11): 183–185
1990	H3N2	[41]; MMWR 1990; 39(17): 293–296
1991	B Virus	[41]; MMWR 1991; 40(14): 231–233, 239–240
1992	H3N2 (+18% H1N1)	[41]; MMWR 1992; 41(18); 315–317, 323
1993	B Virus	[41]; MMWR 1992; 41(18): 315–317, 323

## Supplementary Materials

The following are available online at http://www.mdpi.com/2076-393X/6/3/39/s1, Figure S1: Decline in HI Titer After Vaccination, Figure S2: Titers from Short and Long Service Subgroups, Figure S3: Amount of IgG from Short and Long Service Subgroups, Figure S4: HI Titers with Years of Service, Figure S5: HI Titers for subjects born after the emergence of the H1N1 strain, Figure S6: HI Titers for Different Birth Cohorts.
