# Supplementary material for: The Effects of Birth Year, Age and Sex on Hemagglutination Inhibition Antibody Responses to Influenza Vaccination
Source: Vaccines (Basel). 2018 Jul 3;6(3):39. doi: 10.3390/vaccines6030039 (PMC6161215; doi:10.3390/vaccines6030039)
Supplement: Supplementary file 1 [file vaccines-06-00039-s001.pdf]

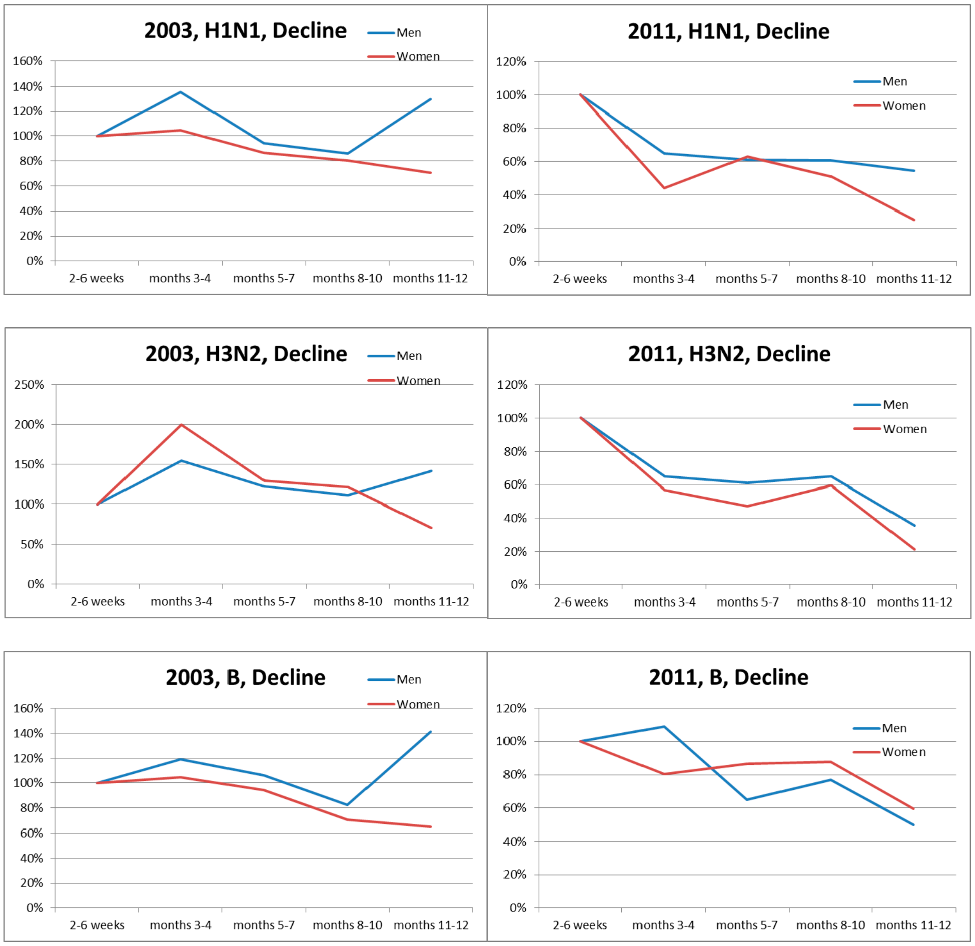

Figure S1. Decline in HI Titer after vaccination.

Geometric mean titers were determined for the second blood draw for each of the binned groups in both the 2003 and 2011 cohorts. The titers are expressed as a percentage of the geometric mean titers from the first blood draw for the same subjects in each binned group. There are 16 men and 16 women in the 3-4 month bin, 24 of each in the 5-7 month bin, 32 of each in the 8-10 month bin, and 16 of each in the 11-12 month bin. The antigens tested with the 2003 cohort were A/New Caledonia/20/1999 (H1N1), A/Moscow/10/1999 (H3N2) and B/Hong Kong/330/2001 (Victoria lineage). The antigens tested with the 2011 cohort were A/California/07/2009 (pdmH1N1), A/Perth/16/2009 (H3N2) and B/Brisbane/60/2008 (Victoria lineage). The values for each time point for men and women are not statistically different (Mann Whitney test).

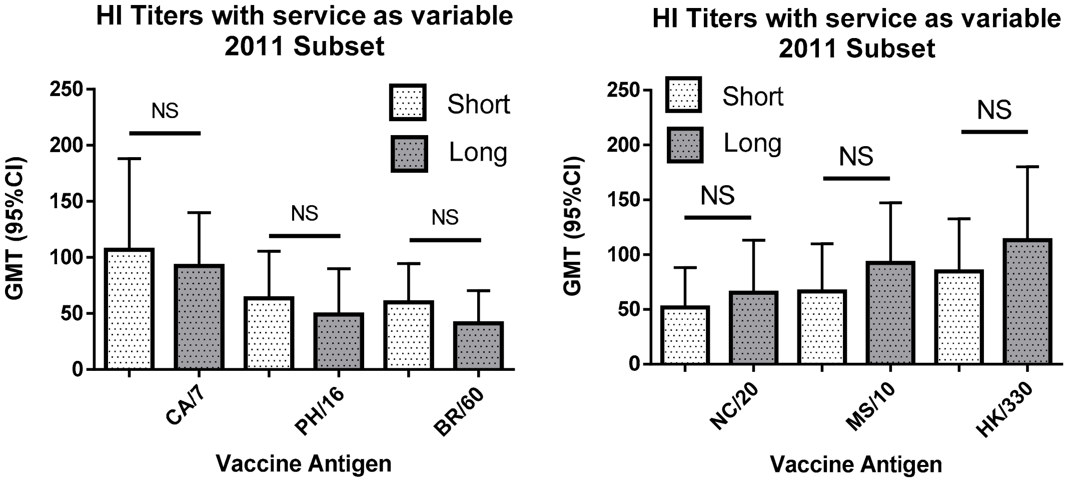

Figure S2. Titers from Short and Long Service Subgroups.

The geometric mean titers (with 95% confidence interval) for sera from personnel with short and long service are shown. The data are from 12 men and 12 women with 8-9 years of service, and 12 of each with 1-3 years of service. The viruses used for testing HI titer are listed on the x axis. Those matching the vaccine antigens are CA/7, A/California/07/2009 (pdmH1N1); PH/16, A/Perth/16/2009 (H3N2); and BR/60, B/Brisbane/60/2008 (Victoria lineage). Those matching antigens that circulated earlier in the subject's lifetime are NC/20, A/New Caledonia/20/1999 (H1N1); MS/10, A/Moscow/10/1999 (H3N2); and HK/330, B/Hong Kong/330/2001 (Victoria lineage). The difference between the short and long service groups was evaluated using the Mann Whitney rank test. n.s., not significant.

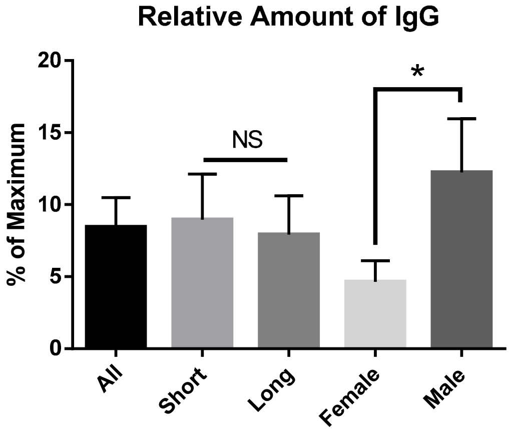

Figure S3. Amount of IgG from Short and Long Service Subgroups.

The amount of IgG for 24 samples from the 2011 cohort (12 short service and 12 long service subjects) were determined as a percentage of a high IgG concentration serum sample. Bars show the mean with the standard error for all samples and subgroups. The significance of the difference between the service groups and between the sexes was evaluated using the Mann Whitney rank test: n.s., not significant; *, $p < 0.05$.

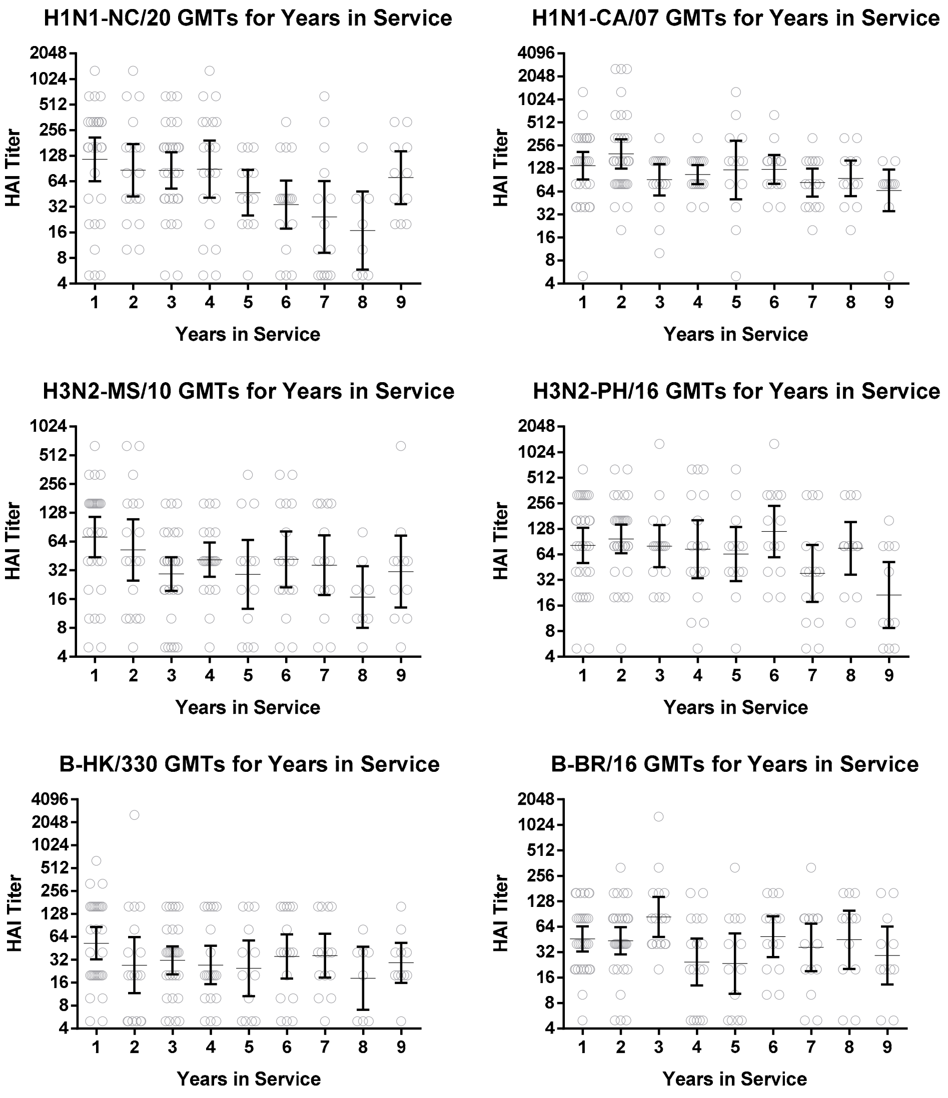

Figure S4. HI Titers with Years of Service.

The geometric mean titers (with 95% confidence intervals) are shown with the number of years in service for the 2003 cohort on the left and the 2011 cohort on the right. The viruses used for testing HI titer are A/New Caledonia/20/1999 (H1N1), A/Moscow/10/1999 (H3N2), and B/Hong Kong/330/2001 (Victoria lineage) for the 2003 cohort. The viruses used for testing HI titer are A/California/07/2009 (pdmH1N1), A/Perth/16/2009 (H3N2), and B/Brisbane/60/2008 (Victoria lineage) for the 2011 cohort.

Some of the higher HAI titers correlate with changes in the virus circulating around the year of birth and some changes correlated with changes in viruses circulating in the years preceding vaccination. There is a drop in H1N1 titers for people with 5-8 years of service compared to those with 1-4 years service for the 2003 cohort. The longer the members were in the service the more likely they were born before the re-emergence of the H1N1 strain in 1977 and this is analyzed further in figure 5 and figure S5. For the 2011 cohort there are slightly higher H1N1 GMTs for those with 2 years of service, higher B titers for those with 3 years of service, and higher H3N2 titers for those with 6 and 8 years of service. These coincide with an H1N1 pandemic two years prior to vaccination in 2009

(https://www.cdc.gov/flu/pastseasons/0910season.htm), a mismatched B vaccine strain when most infections late in the 2007/08 season were B viruses (https://www.cdc.gov/flu/pastseasons/0708season.htm), a mismatched H3N2 season for 2003/04 (https://www.cdc.gov/flu/weekly/weeklyarchives2003-2004/03-04summary.htm), and the 2001-02 season was also an H3N2 dominant season with some H1N2 viruses identified (https://www.cdc.gov/flu/weekly/weeklyarchives2001-2002/01-02summary.htm).

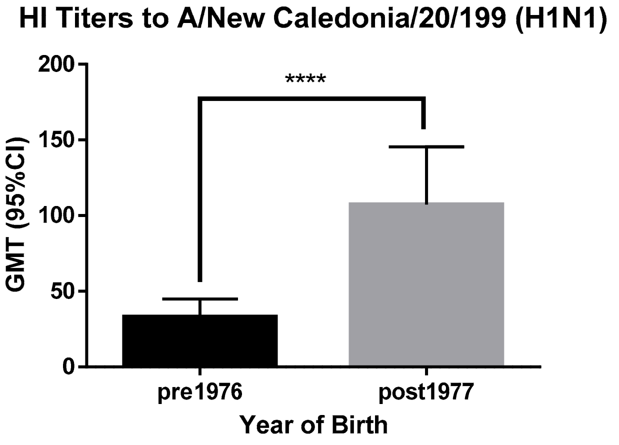

Figure S5. HI Titers for subjects born after the emergence of the H1N1 strain

The geometric mean titers (with confidence interval) for the 2003 cohort based on birth year. The difference between the two groups was evaluated using the Mann Whitney rank test. ****, $p < 0.0001$.

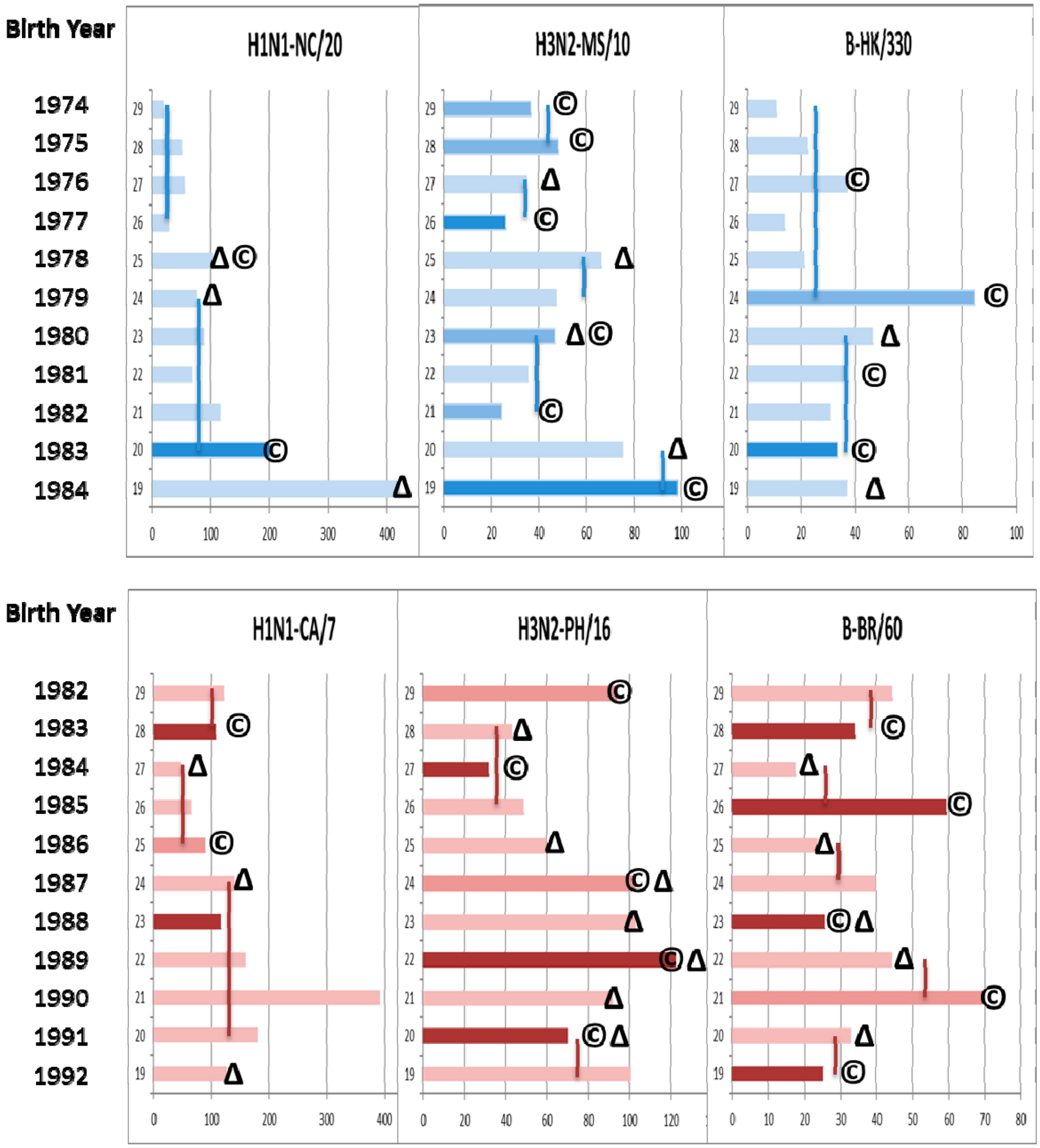

Figure S6. HI Titers for Different Birth Cohorts

The geometric mean titers for the 19-29 year old subjects in each cohort are shown as horizontal bars. The year of birth and age at vaccination are shown on the y axis. The viruses used for testing HI titer are: NC/20, A/New Caledonia/20/1999 (H1N1); MS/10, A/Moscow/10/1999 (H3N2); HK/330, B/Hong Kong/330/2001 (Victoria lineage); CA/7, A/California/07/2009 (pdmH1N1); PH/16, A/Perth/16/2009 (H3N2); and BR/60, B/Brisbane/60/2008 (Victoria lineage). © indicates that the dominant type of virus that circulated the season following the birth year. Vertical bars indicate the geometric mean titer toward the vaccine antigen for all of the years that the circulating virus of that type was antigenically similar (as determined by antigen recommended for vaccine formulation in birth year). Δ indicates a change in the virus strain recommended for vaccines for the birth year (see Figure 5 for statistical analysis). Darker shading of the horizontal bars indicates a more severe influenza season.

Studyid	Year_vaccination	IMM_TYPE	sex	age	Post1_d_event	Post2_d_event	deploy_num	yrs_service	prior_vaccination	S1 H1N1-NC/20	S1 H3N2-MS/10	S1 B-HK/330	S2 H1N1-NC/20	S2 H3N2-MS/10	S2 B-HK/330
WW 005	2003	015	M	22	29	123	1	3		640	80	40	640	160	80
WW 024	2003	015	M	30	31	303	0	2		160	10	2560	160	40	2560
WW 030	2003	015	M	29	39	140	0	9		40	10	5	80	20	10
WW 045	2003	015	M	22	22	226	0	4		160	40	80	5	10	20
WW 046	2003	015	M	29	42	91	0	5	015	80	160	5	40	80	5
WW 049	2003	015	M	37	40	143	0	7		20	20	10	40	20	10
WW 052	2003	015	M	21	23	124	0	1		320	20	160	320	160	160
WW 055	2003	015	M	21	25	211	1	2	015	80	80	20	80	40	20
WW 057	2003	015	M	22	33	132	1	2		10	40	20	5	40	20
WW 062	2003	015	M	27	22	304	0	5		40	5	80	80	5	80
WW 068	2003	015	M	32	40	315	1	7	015	40	160	40	40	40	80
WW 085	2003	015	M	30	33	115	1	9		20	10	20	40	10	20
WW 088	2003	015	M	32	42	215	0	9		160	40	20	160	40	20
WW 091	2003	015	M	34	33	231	0	5	015	20	40	10	40	40	5
WW 093	2003	015	M	20	25	227	0	1	015	80	160	640	80	160	320
WW 096	2003	015	M	41	27	141	0	9		80	20	20	5	20	5
WW 104	2003	015	M	19	32	324	0	1		640	80	80	640	40	80
WW	2003	015	M	2	31	219	1	1	015	20	80	20	20	40	5

107				5											
WW 108	2003	015	M	32	42	122	0	9		80	20	40	160	80	40
WW 115	2003	015	M	26	27	308	1	1	015	10	80	20	40	320	160
WW 122	2003	015	M	32	29	302	0	8	015	40	5	40	40	80	20
WW 124	2003	015	M	24	42	215	1	6		40	160	160	40	160	160
WW 128	2003	015	M	24	32	93	1	4	015	5	80	40	20	320	40
WW 129	2003	015	M	23	28	326	0	3	015	40	40	80	40	20	80
WW 134	2003	015	M	26	35	212	1	4		80	40	160	160	80	80
WW 139	2003	015	M	29	31	308	0	7	015	5	10	20	5	10	40
WW 142	2003	015	M	22	35	121	0	3	015	320	10	40	640	10	80
WW 147	2003	015	M	31	35	217	0	8		10	80	40	10	40	20
WW 151	2002	015	M	29	37	214	1	3		5	40	40	40	40	40
WW 155	2003	015	M	25	42	128	0	4		320	80	5	320	40	5
WW 162	2003	015	M	31	40	108	0	7	015	40	40	40	80	40	80
WW 163	2003	015	M	34	29	113	0	9		20	5	20	40	5	40
WW 164	2003	015	M	33	37	147	1	4		5	20	5	5	40	20
WW 168	2003	015	M	35	26	124	0	6		40	80	40	80	160	40
WW 169	2003	015	M	25	27	215	0	7		80	80	20	40	160	10
WW 178	2003	015	M	19	40	228	1	1		320	40	80	320	160	160
WW 186	2003	015	M	29	30	213	1	3	015	40	40	10	160	40	40
WW	2003	015	M	2	41	212	0	4		80	40	10	5	320	5

187				3											
WW 188	2003	015	M	27	34	139	0	9		320	640	80	160	320	80
WW 191	2003	015	M	27	27	226	0	8	015	160	10	80	160	20	80
WW 192	2003	015	M	21	29	212	1	3		80	40	5	80	40	5
WW 194	2003	015	M	24	29	124	1	4		640	80	160	1280	320	160
WW 195	2003	015	M	30	35	213	0	5	015	80	40	160	80	20	160
WW 196	2003	015	M	28	33	91	0	9	015	320	40	40	320	80	80
WW 202	2003	015	M	20	41	97	0	1		320	40	10	320	320	5
WW 204	2003	015	M	22	38	211	0	3		20	160	40	20	80	20
WW 205	2003	015	M	22	21	95	0	3	015	80	80	160	40	160	160
WW 207	2003	015	M	33	31	143	0	7		5	40	10	20	80	10
WW 211	2003	015	M	25	38	232	0	5	015	160	10	40	80	80	40
WW 216	2003	015	M	20	27	128	0	2	015	40	10	20	160	40	40
WW 218	2003	015	M	26	25	96	0	5		20	20	5	20	160	5
WW 221	2003	015	M	28	35	231	0	6		40	320	80	40	320	80
WW 232	2003	015	M	22	32	117	0	4	015	20	40	20	20	20	20
WW 235	2003	015	M	25	25	108	0	6	015	40	40	40	40	40	80
WW 236	2003	015	M	35	35	112	0	7		5	20	5	5	10	5
WW 238	2003	015	M	28	38	121	0	3	015	40	20	5	40	20	5
WW 242	2003	015	M	27	29	119	1	4		80	10	20	160	10	40
WW	2003	015	M	2	29	115	0	4	015	320	40	20	640	40	10

246				3											
WW 247	2003	015	M	20	33	147	1	1		320	160	20	160	80	20
WW 248	2003	015	M	24	34	92	1	3	015	40	40	10	40	160	20
WW 250	2003	015	M	26	32	230	0	3	015	80	20	20	160	20	10
WW 254	2003	015	M	24	27	97	0	2		160	40	20	640	80	20
WW 260	2003	015	M	20	35	162	1	2		160	80	20	320	40	80
WW 262	2003	015	M	21	37	212	1	3	016	80	80	20	320	160	40
WW 264	2003	015	M	35	33	223	0	8		5	20	20	5	20	10
WW 265	2003	015	M	23	34	212	1	2		80	20	40	80	160	80
WW 267	2003	015	M	25	33	122	1	3	015	320	80	80	320	80	20
WW 269	2003	015	M	31	35	212	1	2		40	160	40	20	80	40
WW 271	2003	015	M	27	24	141	0	9		40	40	40	80	80	40
WW 273	2003	015	M	19	23	142	0	1		640	40	80	640	40	160
WW 275	2003	015	M	20	28	143	0	2		640	320	160	160	320	160
WW 280	2003	015	M	20	21	130	1	2		1280	640	80	80	80	10
WW 281	2003	015	M	21	34	142	1	2		640	640	160	320	320	160
WW 290	2003	015	M	19	26	216	0	1	015	640	80	160	160	20	80
WW 295	2003	015	M	27	33	212	1	9	015	160	80	20	40	20	5
WW 296	2003	015	M	23	33	212	1	5		80	20	20	40	5	5
WW 301	2003	015	M	22	28	216	0	1	088	5	10	10	5	10	40
WW	2003	015	M	3	33	235	0	1		20	10	5	20	20	40

303				3											
WW 307	2003	015	M	21	34	212	1	3		160	20	80	160	80	80
WW 317	2003	015	M	24	35	223	0	3	015	20	20	160	20	40	80
WW 004	2003	015	F	23	31	156	1	1		160	320	160	160	160	160
WW 159	2003	015	F	30	21	363	0	3		40	160	40	40	160	40
WW 073	2003	015	F	29	28	197	0	6		10	20	5	10	20	5
WW 027	2003	015	F	23	29	238	0	3	015	160	20	160	640	10	160
WW 283	2003	015	F	28	42	105	0	4		160	40	10	160	160	20
WW 081	2003	015	F	37	37	176	0	6		20	5	20	20	5	20
WW 002	2003	015	F	21	34	140	0	1		160	5	160	160	5	160
WW 320	2003	015	F	20	29	231	1	2		160	40	5	160	20	5
WW 259	2003	015	F	21	32	182	1	3	015	160	5	5	160	40	5
WW 158	2003	015	F	28	34	324	0	3	015	320	40	40	320	160	40
WW 060	2003	015	F	31	30	351	1	4		40	40	20	10	10	5
WW 300	2003	015	F	30	35	107	1	6	015	40	160	5	40	160	5
WW 131	2003	015	F	31	31	293	0	6		160	20	160	160	40	40
WW 200	2003	015	F	34	29	297	0	1		20	10	160	10	10	40
WW 047	2003	015	F	21	28	229	0	1		160	5	20	40	5	5
WW 037	2003	015	F	40	42	199	0	6		160	20	10	40	5	5
WW 245	2003	015	F	19	29	309	0	1		1280	640	40	640	320	20
WW	2003	015	F	2	27	245	1	1	015	40	20	20	20	40	20

305				5											
WW 117	2003	015	F	32	42	138	0	6		5	10	40	5	5	40
WW 184	2003	015	F	27	27	332	1	4		10	160	5	20	160	5
WW 064	2003	015	F	32	27	310	0	7		160	160	160	40	160	160
WW 297	2003	015	F	24	34	288	1	4		1280	5	160	1280	5	40
WW 240	2003	015	F	24	30	98	1	7	015	320	160	160	320	160	160
WW 042	2003	015	F	22	36	328	0	4		160	160	20	160	20	5
WW 277	2003	015	F	26	25	228	1	8		5	10	5	5	10	5
WW 118	2003	015	F	29	37	302	0	1		5	160	20	5	160	20
WW 039	2003	015	F	22	21	175	0	3	015	40	5	40	20	5	40
WW 261	2003	015	F	31	28	264	0	4		40	20	20	20	40	20
WW 256	2002	015	F	30	22	264	1	7	015	10	20	40	5	40	40
WW 146	2003	015	F	26	31	176	0	6	015	40	20	10	160	640	20
WW 084	2003	015	F	30	38	110	0	7	016	5	5	40	10	160	40
WW 111	2003	015	F	34	23	114	0	7		5	5	160	5	40	160
WW 152	2003	015	F	34	23	169	1	8	016	20	10	5	10	5	5
WW 154	2003	015	F	34	40	146	0	2		10	10	5	5	20	5
WW 270	2003	015	F	24	29	223	0	6	015	5	40	40	10	160	20
WW 028	2003	015	F	19	25	211	1	1	015	320	320	20	640	640	10
WW 125	2003	015	F	29	36	252	1	3	016	160	40	10	160	40	10
WW	2003	015	F	2	41	278	0	4		10	20	160	5	160	5

165				4											
WW 007	2003	015	F	28	41	196	0	8		40	40	40	160	160	40
WW 035	2003	015	F	28	29	255	0	5		40	5	20	20	20	5
WW 291	2003	015	F	20	39	242	1	3		640	5	5	640	5	20
WW 053	2003	015	F	24	22	153	1	4		640	40	160	640	160	160
WW 153	2003	015	F	30	25	264	0	7		640	160	160	640	160	160
WW 103	2003	015	F	28	35	118	0	2		40	160	5	160	160	5
WW 083	2003	015	F	20	29	105	0	2		20	40	5	20	40	5
WW 021	2003	015	F	23	34	291	0	5	015	160	160	160	40	160	160
WW 289	2003	015	F	23	22	106	0	3		640	40	160	320	10	20
WW 222	2003	015	F	34	42	168	0	7		10	40	20	5	40	20
WW 015	2003	015	F	25	35	291	0	3		160	160	40	160	160	20
WW 063	2003	015	F	20	21	162	0	1		160	160	160	160	160	160
WW 171	2003	015	F	27	39	120	0	6		320	40	160	640	320	160
WW 056	2003	015	F	28	34	252	0	6	015	5	320	40	5	320	20
WW 098	2003	015	F	23	28	95	0	1		5	160	20	5	160	20
WW 101	2003	015	F	26	22	93	0	8		5	20	5	40	160	5
WW 279	2003	015	F	36	22	101	0	9	015	20	40	160	10	40	160
WW 137	2003	015	F	28	34	126	0	6		20	5	5	40	5	5
WW 072	2003	015	F	27	34	98	1	2	015	20	10	5	40	20	5
WW	2003	015	F	2	21	92	0	5	015	20	40	40	5	40	40

313				3											
WW 176	2003	015	F	20	42	173	1	1	088	320	160	160	640	320	160
WW 094	2003	015	F	25	25	93	1	5	015	160	320	5	160	160	5
WW 048	2003	015	F	26	34	299	0	4	015	320	40	20	640	160	40
WW 043	2003	015	F	25	24	113	0	1	015	40	40	40	5	320	160
WW 132	2003	015	F	19	33	202	1	1		320	160	20	320	40	5
WW 217	2003	015	F	21	37	280	1	3		5	5	20	20	5	20
WW 029	2003	015	F	35	28	264	0	6		40	40	160	10	20	40
WW 011	2003	015	F	23	39	233	1	5	015	40	5	5	20	5	5
WW 243	2003	015	F	25	22	126	1	4		320	160	10	320	40	40
WW 023	2003	015	F	31	31	299	1	3	015	20	5	40	20	160	160
WW 100	2003	015	F	28	31	191	0	2	015	40	160	160	40	160	160
WW 276	2003	015	F	18	22	169	0	1		320	160	320	160	160	320
WW 054	2003	015	F	19	36	203	0	2		320	5	5	160	160	5
WW 203	2003	015	F	19	33	176	1	1		320	160	160	160	20	40
WW 166	2003	015	F	21	21	133	1	3		160	40	20	160	40	10
WW 006	2003	015	F	19	27	279	0	1		160	320	5	160	320	20
WW 119	2003	015	F	27	30	219	1	5		5	40	160	5	40	160
WW 258	2003	015	F	23	31	299	1	1		320	160	160	160	160	40
WW 038	2003	015	F	22	41	224	0	3	015	160	40	160	40	160	20
WW	2003	015	F	3	38	251	0	1	015	160	160	320	40	40	160

284				3											
WW013	2003	015	F	22	38	241	1	3		160	20	20	40	5	20
WW014	2003	015	F	24	30	224	0	6		160	160	160	160	40	160

Studyid	Year_vaccination	IMM_TYPE	sex	age	Post1_d_event	Post2_d_event	deploy_num	yrs_service	prior_vaccination	S1 H1N1-CA/7	S1 H3N2-PH/16	S1 B-BR/60	S2 H1N1-CA/7	S2 H3N2-PH/16	S2 B-BR/60
WW009	2011	141	M	32	25	140	0	8	015	320	320	160	80	80	80
WW016	2011	141	M	28	23	233	1	7	015	160	80	80	160	20	80
WW036	2011	141	M	22	39	211	1	3	111	160	80	20	160	80	40
WW061	2011	141	M	25	42	130	1	1	111	40	20	80	20	10	40
WW071	2011	141	M	26	34	220	0	1		80	20	80	80	40	40
WW076	2011	140	M	21	29	92	0	2	111	640	160	40	640	80	20
WW079	2011	140	M	38	28	138	0	9	015	160	160	160	160	320	160
WW089	2011	141	M	29	27	306	0	9	111	40	5	20	40	5	20
WW090	2011	140	M	20	31	56	0	1	111	80	5	5	80	5	5
WW095	2011	140	M	23	35	94	1	2	111	80	640	160	80	640	160
WW099	2011	140	M	27	22	50	0	7	015	40	10	40	40	10	20
WW52	2011	140	M	25	21	91	0	4	111	160	80	80	160	160	80
WW59	2011	141	M	27	33	124	1	8	111	160	80	80	160	40	80
WW113	2011	141	M	29	35	150	0	3	015	40	80	80	40	160	40

WW114	2011	141	M	20	24	91	0	2	141	160	20	20	160	40	40
WW127	2011	015	M	20	22	212	0	2	015	2560	640	80	1280	320	20
WW130	2011	140	M	25	40	126	0	3	015	160	80	40	160	160	80
WW133	2011	140	M	23	24	136	0	4	015	80	5	20	160	5	80
WW136	2011	141	M	25	21	217	0	5	015	160	80	40	160	80	40
WW140	2011	141	M	29	32	95	1	7	111	160	320	20	80	160	20
WW141	2011	141	M	29	29	218	0	7	015	160	80	20	80	40	20
WW144	2011	141	M	28	35	98	0	8	111	40	10	5	80	10	5
WW145	2011	140	M	29	33	126	0	4	111	160	160	160	160	40	160
WW150	2011	141	M	23	28	306	1	5	111	1280	640	80	640	160	20
WW156	2011	141	M	22	23	121	1	1	111	320	320	160	40	80	20
WW157	2011	141	M	27	35	124	0	4	111	80	20	40	20	40	40
WW160	2011	140	M	32	29	212	0	4	015	160	40	5	160	80	5
WW161	2011	140	M	20	41	138	0	2	015	80	40	20	160	40	10
WW167	2011	141	M	23	30	224	0	5	088	40	40	5	40	80	5
WW170	2011	141	M	24	34	212	1	3	111	80	160	40	80	80	40
WW172	2011	140	M	25	27	121	1	7	111	40	5	5	40	5	5
WW173	2011	141	M	29	25	326	0	4	015	80	40	80	20	10	40
WW175	2011	141	M	38	35	365	1	3		80	1280	1280	40	320	320
WW179	2011	141	M	21	37	217	0	2	111	160	80	80	160	40	5

WW180	2011	141	M	31	23	212	1	3	111	160	320	160	5	80	80
WW183	2011	140	M	34	22	139	0	7	015	40	20	10	20	5	5
WW193	2011	140	M	37	36	225	0	3	015	160	20	160	80	40	160
WW197	2011	141	M	21	41	127	1	2	111	160	40	10	160	80	5
WW199	2011	141	M	32	39	238	0	3		160	40	40	80	5	40
WW206	2011	141	M	37	40	322	1	6	111	80	1280	80	80	1280	160
WW208	205	015	M	21	42	217	0	2	111/127	640	160	160	640	320	160
WW209	2011	015	M	24	24	305	0	2		640	80	40	320	40	20
WW25	2011	141	M	26	22	56	1	1	111	320	40	20	320	10	20
WW212	2011	141	M	27	42	214	1	2	111	80	20	20	40	5	20
WW213	2011	141	M	20	26	212	1	1	111	320	320	40	80	160	20
WW214	2011	141	M	26	38	122	0	4	015	40	10	160	40	10	160
WW215	2011	141	M	31	31	123	1	5	111	320	40	80	320	40	80
WW223	2011	141	M	27	35	217	0	5	015	5	5	5	5	5	5
WW224	2011	141	M	24	42	98	1	2	111	160	160	40	160	160	80
WW225	2011	140	M	19	32	130	0	1	111	160	40	40	160	20	20
WW226	2011	140	M	32	40	214	1	9	015	80	5	20	80	5	20
WW227	2011	141	M	40	27	58	1	4		160	640	40	5	5	5
WW228	2011	141	M	29	21	91	1	8	015	80	80	40	80	80	80
WW229	2011	140	M	28	39	312	0	1	016	160	80	40	80	5	10

WW230	2011	141	M	20	34	301	0	2		160	20	20	80	10	10
WW231	2011	141	M	20	40	124	0	1	111	320	160	20	160	40	10
WW233	2011	141	M	22	32	131	0	4	015	80	80	80	80	40	80
WW234	2011	141	M	22	33	134	1	2	111	160	320	160	80	40	40
WW237	2011	141	M	37	23	50	0	8	141	160	320	80	20	80	40
WW239	2011	141	M	25	28	98	0	4	015	160	10	20	160	10	40
WW249	2011	141	M	22	29	97	0	4		160	320	5	80	320	40
WW251	2011	140	M	28	22	214	0	6		80	80	80	10	40	10
WW252	2011	141	M	29	33	124	1	7		160	320	80	80	80	20
WW255	205	015	M	27	22	215	0	9	015/127	5	10	5	160	5	5
WW257	2011	015	M	25	40	126	0	5		160	40	20	40	20	5
WW266	2011	140	M	39	24	297	0	7	015	160	40	80	160	40	20
WW272	2011	141	M	22	32	222	1	2		1280	160	40	1280	320	40
WW274	2011	141	M	32	42	127	1	6		160	80	160	40	80	320
WW282	2011	141	M	24	29	56	0	2	015	160	80	5	80	160	10
WW285	2011	141	M	35	26	136	1	3		80	20	40	10	5	10
WW293	2011	140	M	40	39	237	0	2		320	80	320	160	20	80
WW294	205	015	M	23	23	225	0	2	111/127	40	160	40	5	80	80
WW302	2011	141	M	27	24	223	1	5	111	160	160	10	80	40	80
WW304	2011	140	M	38	31	223	1	6	111	160	320	160	40	320	160

WW306	2011	140	M	31	36	218	0	9	015	80	80	160	20	80	160
WW308	2011	140	M	19	40	215	0	1	015	160	320	80	160	320	80
WW312	2011	141	M	35	42	278	0	9		160	40	40	80	20	40
WW315	2011	141	M	40	22	236	1	9	015	80	80	80	80	20	80
WW316	2011	141	M	21	35	215	1	2	111	320	20	40	80	5	20
WW318	2011	140	M	22	24	211	0	4	088	160	40	5	80	40	5
WW292	2011	140	F	33	21	162	0	8	015	160	80	160	80	40	160
WW126	2011	141	F	29	34	216	1	6	015	640	320	80	640	320	160
WW123	2011	141	F	22	40	271	1	1	111	80	80	160	80	5	40
WW244	2011	141	F	24	24	136	1	2		80	80	40	40	40	5
WW059	2011	141	F	26	25	227	0	5	015	320	80	80	320	80	80
WW253	2011	141	F	21	35	110	0	1		320	160	160	160	80	80
WW241	2011	140	F	37	41	167	0	2		160	80	40	160	80	40
WW181	2011	140	F	29	22	310	0	7	111	320	40	320	160	20	160
WW198	2011	140	F	19	27	119	0	1		320	320	20	80	80	5
WW110	2011	141	F	23	29	106	1	4		320	640	5	5	320	5
WW143	2011	141	F	27	35	105	0	5		80	320	20	80	320	40
WW032	2011	141	F	25	36	112	0	6	111	160	40	40	80	5	40
WW149	2011	140	F	27	41	157	1	9		80	80	20	40	80	20
WW018	2011	140	F	30	32	139	0	1	015	160	320	160	20	80	160

WW033	2011	140	F	19	38	109	0	1	015	40	80	20	20	40	20
WW074	2011	140	F	20	41	291	0	2	111	320	80	160	40	20	20
WW051	2011	140	F	25	28	154	0	1	111	80	160	20	40	80	20
WW092	2011	141	F	23	42	140	0	5	015	20	40	40	10	40	40
WW065	2011	141	F	25	40	218	0	4		80	640	40	80	320	40
WW288	2011	141	F	28	40	113	1	8	015	80	20	20	80	40	40
WW058	2011	141	F	30	27	300	0	6		160	320	20	80	160	20
WW190	2011	140	F	28	22	115	0	8	015	320	80	160	160	40	40
WW070	2011	141	F	28	35	127	0	4	015	80	80	20	40	40	20
WW020	2011	141	F	24	32	303	1	3	015	80	160	80	10	40	20
WW105	2011	141	F	23	29	166	1	1	111	1280	80	40	1280	40	40
WW003	2011	141	F	26	21	171	0	2	015	20	160	80	5	40	80
WW050	2011	141	F	33	33	250	0	6	111	160	20	20	160	10	10
WW174	2011	140	F	21	34	162	0	1	015	640	20	40	160	5	20
WW044	2011	140	F	23	34	237	0	5	111	80	80	5	160	20	5
WW077	2011	141	F	24	37	232	1	4	111	80	40	5	5	5	5
WW041	2011	141	F	25	29	125	1	6		40	80	10	40	40	20
WW112	2011	141	F	30	26	307	0	7	088	40	40	80	20	5	160
WW040	2011	141	F	39	26	305	1	2	111	80	160	5	5	5	5
WW075	2011	140	F	22	26	223	0	3		320	40	320	160	10	160

WW1 82	2011	141	F	3 1	28	224	1	7	015	80	320	80	80	320	40
WW0 31	2011	141	F	3 3	23	134	0	3		10	20	80	5	5	80
WW0 82	2011	140	F	3 6	36	254	0	9		80	5	5	5	40	10
WW1 77	2011	141	F	2 2	35	123	1	2		80	80	40	80	40	40
WW2 20	2011	141	F	3 3	28	268	0	8	015	20	20	80	10	5	80
WW0 69	2011	141	F	3 7	41	304	1	7	111	80	40	80	20	10	40
WW0 19	2010	015	F	2 0	34	288	0	2	127	80	80	40	80	160	80
WW0 78	2011	015	F	2 3	32	348	0	3		160	80	40	160	80	20
WW0 86	2011	141	F	2 7	35	107	1	6	111	80	320	40	160	320	40
WW1 21	2011	140	F	2 6	28	272	1	3	111	20	80	80	20	160	80
WW3 19	2011	141	F	2 0	41	243	1	1	111	160	640	80	80	320	40
WW1 06	2011	141	F	2 5	40	168	0	2	015	40	320	5	20	160	5
WW2 63	2011	140	F	3 1	29	146	1	9	015	80	10	40	80	10	40
WW0 34	2011	140	F	2 7	42	293	0	7		20	10	20	40	10	40
WW1 35	2011	141	F	2 4	22	120	1	1	111	160	320	80	320	160	40
WW2 78	2011	140	F	1 9	42	161	0	1		40	20	10	40	5	5
WW2 99	2011	141	F	3 2	30	293	1	2	088	320	160	80	80	80	40
WW3 09	2011	140	F	4 0	41	100	1	6		320	160	80	20	320	160
WW3 11	2011	141	F	2 9	37	114	1	2	111	320	320	80	160	80	80
WW2 19	2011	140	F	2 8	29	340	0	9		80	10	20	5	5	20

WW087	2011	141	F	19	27	315	0	1		320	160	20	160	10	5
WW148	2011	140	F	21	22	129	0	1	111	160	160	160	160	5	40
WW010	2011	141	F	22	30	204	0	2	015	80	320	80	160	320	160
WW201	2011	141	F	23	32	121	1	1	111	40	80	40	20	40	20
WW314	2011	141	F	38	36	116	0	2	111	80	5	80	40	10	40
WW012	2011	141	F	26	31	107	0	8		40	80	20	10	20	10
WW067	2011	140	F	22	36	94	0	1		320	40	20	160	20	20
WW080	2011	140	F	29	37	295	0	8		80	320	5	20	320	5
WW097	2011	141	F	29	26	125	1	6		40	20	10	20	10	10
WW001	2010	015	F	27	32	257	0	7	015/127	80	5	5	20	40	20
WW025	2011	141	F	24	26	196	0	5	015	160	80	320	40	20	80
WW310	2011	140	F	40	33	264	0	6		160	160	160	160	80	320
WW189	2011	140	F	21	26	234	1	1		320	320	160	80	5	40
WW287	2011	141	F	33	23	168	1	1	015	40	40	80	40	80	160
WW138	2011	140	F	24	33	120	0	1	015	320	160	80	320	80	80
WW185	2011	141	F	34	35	154	1	7	015	80	20	40	320	20	160
WW022	2011	140	F	41	41	247	0	3	015	80	80	160	40	40	80
WW116	2010	141	F	24	29	239	0	6	127	80	40	40	160	40	40
WW026	2011	141	F	28	29	275	1	3	111	160	80	40	80	40	20
WW268	2011	140	F	38	26	241	1	5	015	640	20	5	160	5	20

WW008	2011	141	F	30	39	279	0	8	015	80	80	80	40	40	40
WW120	2011	141	F	20	42	246	0	1	015	40	20	40	10	40	40
WW286	2011	141	F	35	39	253	0	2	111	2560	80	40	320	80	40
WW298	2011	141	F	41	33	234	1	1	111	5	5	20	5	5	20
WW066	2011	140	F	21	38	249	1	2	111	2560	160	80	1280	80	20
WW017	2011	140	F	22	34	258	0	4		40	320	20	40	320	80
